# 基于3种非天然糖代谢标记的分泌蛋白质组分析性能对比

**DOI:** 10.3724/SP.J.1123.2021.04017

**Published:** 2021-10-08

**Authors:** Yuan MAO, Jiangnan ZHENG, Shun FENG, Ruijun TIAN

**Affiliations:** 1.西南交通大学生命科学与工程学院, 四川 成都 610031; 1. School of Life Science and Engineering, Southwest Jiaotong University, Chengdu 610031, China; 2.南方科技大学理学院化学系, 广东 深圳 518055; 2. Department of Chemistry, School of Science, Southern University of Science and Technology, Shenzhen 518055, China

**Keywords:** 液相色谱, 串联质谱, 分泌蛋白, 点击化学, 代谢标记, 糖类似物, liquid chromatography (LC), tandem mass spectrometry (MS/MS), secreted protein, click chemistry, metabolic labeling, sugar analogues

## Abstract

分泌蛋白质是调控细胞间信号转导的重要生物大分子。由于分泌蛋白的丰度相比于胞内蛋白以及培养基添加剂更低,因此分泌蛋白的高通量鉴定是目前蛋白质组学界研究的热点和难点。目前,基于生物质谱的分泌蛋白质组学分析一般均需要从无血清的条件培养基中获得分泌蛋白质,再对其进行富集和分析。该流程操作步骤繁琐,易造成分泌蛋白质的损失和降解。本工作采用基于生物正交化学生物学技术实现对分泌蛋白质的高选择性标记和高效富集。通过结合点击化学技术,综合评估了分泌蛋白质分析中用于代谢标记的不同糖类似物。采用3种最常用的商品化糖类似物,*N*-叠氮乙酰甘露糖胺(ManNAz)、*N*-叠氮乙酰半乳糖胺(GalNAz)和*N*-叠氮乙酰葡萄糖胺(GlcNAz)分别对HeLa细胞进行代谢标记,之后通过炔基生物素探针对条件培养基中的分泌蛋白进行富集,结合质谱分析来对比3种糖类似物对分泌蛋白的标记效率。最后通过无标定量蛋白质组学分析,系统评估了3种糖类似物用于分泌蛋白质组分析的性能。结果表明,基于ManNAz的分泌蛋白标记方法鉴定到了282个分泌蛋白、224个细胞质膜蛋白以及846个*N*-糖基化位点;对分泌蛋白的富集效率分别较GalNAz和GlcNAz提高了130%和67.2%;对细胞质膜蛋白的富集效率较GalNAz和GlcNAz分别提高了273.3%和148.7%,体现出了明显的优势。本研究的实验结果为分泌蛋白高选择性富集和系统分析提供了有益的对比分析和新技术策略。

## Abstract (en)

Many secreted proteins, including cytokines, growth factors and hormones, are crucial in processes like intercellular signaling. Dynamic changes in secreted proteins usually reflect the growth and pathological state of the cells. Many drug targets are secretory proteins. The proteins are also important biomarkers. Conditioned cell culture media are important samples for secretory proteomic studies. Biomass spectrometry-based proteomic analysis enables the systematic study of secretory proteins. The main problem in analyzing secretory proteins in conditioned culture media is the low concentration of these proteins and the presence of serum, amino acids, and additives in culture media that may interfere with the protein analysis. Conventional secretory proteome analysis uses serum-free cell culture to reduce sample complexity, and typically involves protein concentration, purification, and desalting using ultrafiltration, dialysis, lyophilization, and trichloroacetic acid (TCA) or acetone precipitation, followed by enzymatic digestion and mass spectrometry analysis. This analytical process does not allow specific enrichment of secreted proteins. Thus, only a few secreted proteins can be identified. In addition, prolonged serum-free incubation of cells also tends to lead to unexpected changes in their activity status. A bioorthogonal-based enrichment approach can effectively avoid this problem. In recent years, unnatural sugars containing bio-orthologous groups, such as azide groups, have been used to metabolically label glycosylated proteins, enabling cellular imaging or selective enrichment of glycoproteins and their use for proteomic analysis. The strategy is a two-step process. First, azide-based sugar analogues are added to the cell culture medium and introduced to glycoproteins via the intracellular glycan biosynthesis pathway. Second, they are specifically covalently labeled with imaging probes or affinity probes via click chemistry. Since secreted proteins are usually glycoproteins, this glycolytic labeling has been used to label and enrich secreted proteins. *N*-Azidoacetylgalactosamine (GalNAz), *N*-azidoacetylglucosamine (GlcNAz), and *N*-azidoacetylmannosamine (ManNAz) are classical azide-based sugar analogues. Their effects on cytoplasmic membrane proteins have been compared. However, only ManNAz has been used for metabolic labeling of secreted proteins. No other glyco-analogues that label secreted proteins have been reported. Here, the bio-orthogonal chemical biology technology achieved highly selective labeling and enriched secreted proteins. In combination with click chemistry, different sugar analogues were evaluated for metabolic labeling of secreted proteins. HeLa cells were metabolically labeled by ManNAz, GalNAz, and GlcNAz (the three most commonly used commercial sugar analogues). These glycolytic markers can selectively label specific types of glycosylation. For example, ManNAz, an analogue of the biosynthetic precursor of sialic acid, *N*-acetylmannosamine (ManNAc), can label sialylated *N*- or *O*-glycoproteins. GalNAz, an analogue of *N*-acetylgalactosamine (GalNAc), can replace GalNAc as a core residue of mucin-type *O*-glycans and thus label *O*-glycoproteins. In addition, the intracellular metabolic intermediate of GalNAz (pyrophosphate) UDP-GalNAz can be interconverted with UDP-GlcNAz catalyzed by UDP-galactose-4-differential isomerase (GALE) and thus can also label *N*-glycoproteins and *O*-GlcNAc glycoproteins instead of GlcNAc. The GlcNAz analogue is commonly used to label nuclear and cytoplasmic glycoproteins with *β*-*O*-GlcNAc residues, but can also label *N*-glycoproteins with mucin-type *O*-glycoproteins by converting GALE to GalNAz, followed by enrichment using a biotin-alkynyl probe. Label-free quantitative proteomic analysis was performed to evaluate their labeling efficiency. ManNAz-based secretory protein labeling identified 282 secretory proteins, 224 plasma membrane proteins, and 846 *N*-glycosites. Compared with GalNAz and GlcNAz, the enrichment of secreted proteins was increased 130% and 67.2%, respectively, and the enrichment of plasma membrane proteins was increased 273.3% and 148.7%, respectively. This study provides a useful comparative analysis and new strategies for highly selective enrichment and systematic secretome analysis.

分泌蛋白质组(secretome)是指细胞、组织等分泌的全部蛋白质,广义的分泌蛋白组还包括从细胞膜表面脱落(ectodomain shedding)的细胞质膜蛋白^[1]^。许多分泌蛋白,如细胞因子、生长因子和激素等,在细胞间信号转导等过程中发挥着关键的功能。分泌蛋白的动态变化通常反映了细胞的生长情况和病理状态。分泌蛋白构成了很大一部分药物靶标,同时也是重要的生物标志物^[2]^。细胞的条件培养基是分泌蛋白质组研究的重要样本。目前,基于生物质谱的蛋白质组学分析可以实现对分泌蛋白的系统研究^[3]^。分析培养基中的分泌蛋白的主要问题在于条件培养基中的分泌蛋白浓度较低^[4,5,6,7]^,培养基中的血清、氨基酸和添加剂等的存在可能会干扰后续的蛋白质分析。常规的分泌蛋白质组分析使用无血清细胞培养以降低样品的复杂性,一般先采用超滤、透析、冻干和三氯乙酸(TCA)或丙酮沉淀进行蛋白质的浓缩、纯化和除盐,然后进行酶解和质谱分析。这一分析流程无法实现特异性的富集分泌蛋白,从而导致分泌蛋白的鉴定数目较少。此外,长时间无血清培养细胞也容易导致其活性状态的意外改变。而基于生物正交的富集方式则可以有效避免这个问题。

近年来,含有生物正交基团(如叠氮基)的非天然糖已被用于代谢标记糖基化蛋白质^[8,9]^,从而实现对糖蛋白的细胞成像或选择性富集并用于蛋白质组学分析。该策略分为两个步骤:叠氮基糖类似物被添加到细胞培养基中,通过细胞内的聚糖生物合成途径引入到糖蛋白上;然后通过点击化学反应特异性地与成像探针或亲和探针进行共价标记。由于分泌蛋白通常是糖蛋白,这种糖代谢标记已被用于分泌蛋白的标记和富集^[10]^。*N*-叠氮乙酰半乳糖胺(GalNAz)、*N*-叠氮乙酰葡萄糖胺(GlcNAz)和*N*-叠氮乙酰甘露糖胺(ManNAz)是最经典的叠氮基糖类似物^[8,11,12]^,有研究对比了它们对细胞质膜蛋白的标记效果,然而,目前只有ManNAz被用来进行分泌蛋白的代谢标记,尚未报道其他两种糖类似物用于标记分泌蛋白。

本文通过结合糖代谢标记、点击化学和基于质谱的蛋白质组学,系统地评估了ManNAz、GalNAz和GlcNAz 3种非天然糖对分泌蛋白的标记情况。通过无标定量(LFQ)蛋白质组学分析,探索最适合的分泌蛋白的糖代谢标记方法。这项工作提供了一种对细胞分泌蛋白进行全局、位点特异性以及定量研究的手段,并为分泌蛋白的糖代谢标记方法的选择提供依据。

## 1 实验部分

### 1.1 仪器、试剂与材料

Easy-nLC 1000色谱系统(Thermo Fisher Scientific);Orbitrap Fusion质谱仪(Thermo Fisher Scientific)。

高糖DMEM(达尔伯克改良伊格尔,dulbecco’s modified eagle’s medium)培养基、磷酸盐缓冲液(PBS)购自Corning公司。胎牛血清(FBS)购自GIBCO公司。GalNAz、GlcNAz、ManNAz、三(3-羟丙基-三唑甲基)胺(THPTA)购自Click Chemistry Tools公司。0.22 μm一次性无菌过滤器、Amicon Ultra超滤管(15 mL,截止相对分子质量3 kD)购自Millipore公司。盐酸氨基胍、五水硫酸铜、三氟乙酸(TFA)购自阿拉丁试剂有限公司。链霉亲和素(streptavidin)琼脂糖微球购自Thermo Scientific。乙二胺四乙酸二钠(EDTA-Na_2_)购自Amresco公司。苯基甲磺酰氟(PMSF)购自Auragene公司。乙腈(ACN,色谱纯)购自BCR国际贸易公司。甲醇(色谱纯)购自Merck公司。十二烷基硫酸钠(SDS)购自Biosharp公司。抗坏血酸钠、氯乙酰胺(CAA)、三羟甲基氨基甲烷(Tris)、碳酸氢铵、尿素、三(2-羧乙基)膦盐酸盐(TCEP)购自Sigma-Aldrich公司。胰蛋白酶(测序级)购自Promega公司。Lys-C(测序级)购自Wako公司。炔基生物素探针由本实验室合成。

### 1.2 实验步骤

1.2.1 细胞培养与代谢标记

将HeLa细胞置于37 ℃、5% CO_2_的加湿培养箱中,在含(10%, v/v)FBS的高糖DMEM培养基中培养。待细胞生长至覆盖培养皿底部面积的60%时,用PBS洗涤细胞3次,然后分别向无血清DMEM培养基中添加0.1 mmol/L ManNAz、GalNAz和GlcNAz进行代谢标记,细胞继续培养24 h后完成代谢标记过程。

1.2.2 条件培养基的收集

收集条件培养基,以300 g的转速离心5 min,取上清液再以3000 g的转速离心10 min,以去除细胞碎片和死细胞。之后收集上清液,用0.22 μm滤膜过滤。在滤液中加入1 mmol/L EDTA-Na_2_、10 mmol/L CAA和0.1 mmol/L PMSF,然后使用3 kDa超滤管于4 ℃、4000 g条件下将条件培养基浓缩至约600 μL。向超滤管中加入12 mL PBS,再将其超滤浓缩至约600 μL,重复此过程3次,使分泌蛋白样品置换到PBS中。使用Pierce 660蛋白测定试剂测定蛋白质浓度。

1.2.3 利用点击化学探针进行铜催化的点击反应

铜(Ⅰ)催化的叠氮化物-炔烃环加成反应(CuAAC)^[13,14]^是应用最广泛的点击反应。将超滤后的样品通过CuAAC点击反应^[15]^与炔基化试剂进行反应。我们在每个样品中依次加入100 μmol/L炔基生物探针、13.5 mmol/L盐酸氨基胍和催化剂(0.675 mmol/L硫酸铜、3.38 mmol/L THPTA和新制的16.9 mmol/L抗坏血酸钠的预混溶液)(均为加入后的浓度),在常温下振荡反应2 h。通过氯仿-甲醇蛋白沉淀法去除多余的探针。之后将蛋白沉淀溶解于尿素缓冲液(8 mol/L尿素,200 mmol/L Tris, pH 7.4)中。

1.2.4 生物素化蛋白的富集与酶解

将1.2.3中得到的蛋白溶液与15 μL Streptavidin琼脂糖微球置于旋转混匀仪上于4 ℃孵育2 h。依次使用0.4 mL尿素缓冲液(6 mol/L尿素、0.1% SDS、10 mmol/L Tris, pH 7.4)和0.4 mL 1 mol/L氯化钠溶液各洗涤3次。然后向样品管中加入300 μL的烷基化反应缓冲液(5 mmol/L TCEP、50 mmol/L CAA、0.2 mol/L碳酸氢铵、0.5 mol/L氯化钠),于37 ℃振荡孵育30 min。之后向样品管中分3次加入0.4 mL的20%(v/v)乙醇水溶液洗涤。微球用胰蛋白酶(酶与底物质量比为1;200)在50 mmol/L碳酸氢铵和37 ℃条件下酶解12 h。将微球悬浊液离心后,收集上清液。之后用PNGase F(酶与底物质量比为1;200)酶解12 h;将微球悬浊液离心后收集上清液。将上清液用三氟乙酸(TFA)酸化至pH<3后使用StageTips进行脱盐。

1.2.5 液相色谱-串联质谱分析

液相色谱柱为C18毛细管填充柱(15 cm×100 μm, 粒径1.9 μm,孔径12 nm, Dr. Maisch公司)。流动相A相为0.1%(v/v)甲酸水溶液;B相为0.1%(v/v)甲酸乙腈溶液。流速为250 nL/min。色谱有效梯度为7%B在100 min提高至22%B; 22%B在100~120 min提高至35%B。质谱使用数据依赖采集模式,循环时间为3 s,一级质谱离子扫描的*m/z*范围为350~1550,分辨率为120000,自动增益控制(AGC)为2×10^5^,最大注入时间(IT)为100 ms,分离窗口1.6 Da,离子价态限定为2~4,动态排除时间为30 s。二级质谱检测器类型为离子阱,碎裂模式为HCD,最大IT为35 ms,归一化碰撞能(NCE)为30。

1.2.6 数据分析

质谱数据使用MaxQuant^[16]^和UniProtKB人类蛋白质组数据库(2020年1月发布,74823个条目,附加188个高丰度牛血清蛋白序列)对质谱原始数据进行检索。酶的特异性设置为Trypsin/P,最多允许两个酶切遗漏点。固定修饰设置为半胱氨酸烷基化,可变修饰设置为甲硫氨酸氧化和天冬酰胺脱酰胺化。LFQ分析使用“Match between runs”功能,蛋白质和多肽鉴定的假阳性率(FDR)控制在1%以内。

使用Perseus软件(1.6.17.0版)^[17]^进行统计分析。过滤掉反库序列、仅靠位点鉴定的蛋白和污染蛋白。鉴定的蛋白质至少在一组(每组3次生物学独立重复)中有>1个unique肽段并且拥有3个有效值的蛋白质数据被保留做定量分析。LFQ强度进行log_2_变换,缺失值依据正态分布替换width(替换区间宽度): 0.3, downshift(替换位置下调度): 1.8。通过双侧*t*检验分析两组之间的差异性(基于置换检验的(permutation-based)FDR<0.05, S0=2)。使用DAVID(6.8版)^[18,19]^进行基因本体(GO)富集分析,分类的*p*值<0.05。对于*N*-糖基化位点分析,仅保留符合N-X-S/T/C(X≠P)序列的肽段,并筛选出位点定位得分≥50、定位概率≥0.8的数据进行分析。

## 2 结果与讨论

### 2.1 实验设计与可行性讨论

糖代谢标记可以选择性地标记特定的糖基化类型(见图1a)^[20]^。例如ManNAz是唾液酸的生物合成前体*N*-乙酰甘露糖胺(ManNAc)的类似物,可以标记唾液酸化的*N*-或*O*-糖蛋白^[20,21]^。GalNAz是*N*-乙酰半乳糖胺(GalNAc)的类似物,可以替代GalNAc成为黏蛋白型*O*-聚糖的核心残基从而标记*O*-糖蛋白^[20,21]^。此外,GalNAz在细胞内的代谢中间体尿苷二磷酸(UDP)-GalNAz可经过UDP-半乳糖-4-差向异构酶(GALE)催化而与UDP-GlcNAz相互转化^[22]^,因此也可替代GlcNAc对*N*-糖蛋白和*O*-GlcNAc糖蛋白进行标记。*N*-乙酰葡萄糖胺(GlcNAc)的类似物GlcNAz通常用于标记带有*β*-*O*-GlcNAc残基的核糖蛋白和细胞质糖蛋白,但是也能通过GALE转化为GalNAz从而标记*N*-糖蛋白与黏蛋白型*O*-糖蛋白^[20,21]^。

**图1:**
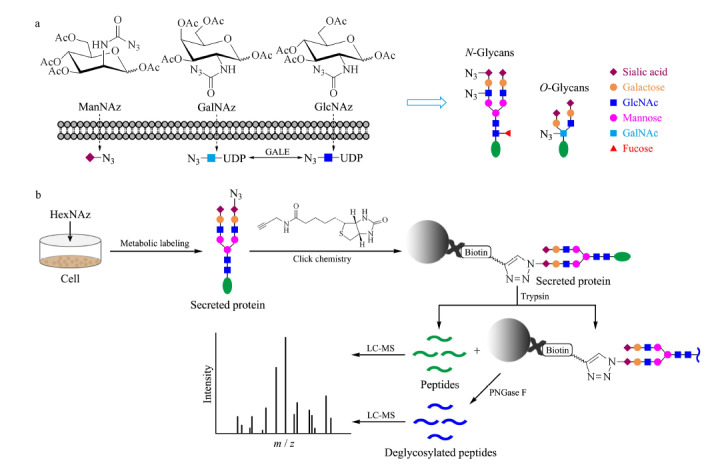
基于质谱分析的分泌蛋白富集

因此,我们选择使用ManNAz、GalNAz和GlcNAz 3种最常用的叠氮基糖类似物对HeLa细胞的分泌蛋白进行代谢标记。收集条件培养基中的叠氮标记的分泌蛋白,通过CuAAC与炔基生物素反应,再用Streptavidin微球富集生物素化的蛋白质。使用胰蛋白酶(trypsin)对富集到的蛋白质进行酶解,再将酶解下来的肽段进行LC-MS检测。之后通过*N*-糖酰胺酶F(PNGase F)酶切释放微球上保留的*N*-糖肽,将得到的去糖基化肽段进行质谱分析,以获得*N*-糖基化位点信息(见图1b)。

### 2.2 不同糖类似物标记的细胞中分泌蛋白的鉴定分析

将基于3种糖类似物标记的方法在3次重复实验中富集到的蛋白质组和特异性肽段(unique peptide)筛选出来进行对比分析。GalNAz组鉴定到了1487个蛋白和11520个特异性肽段;GlcNAz组鉴定到了1462个蛋白和11612个特异性肽段;ManNAz组鉴定到了1591个蛋白以及12187个特异性肽段(见图2a)。进一步对各平行实验进行定量重复性评估,各平行实验间的皮尔逊相关系数都大于0.94,表现出了良好的生物学重复性(见图2b)。3组实验鉴定到的共有蛋白超过了80%。我们将3组实验中鉴定到的蛋白与Uniprot的蛋白数据库进行对比,结果表明ManNAz组检测到的1591个蛋白中有282个蛋白被Uniprot注释为分泌蛋白,224个蛋白被注释为细胞质膜蛋白;GalNAz组鉴定到的1487个蛋白中有271个蛋白被注释为分泌蛋白,204个蛋白被注释为细胞质膜蛋白;GlcNAz组鉴定到的1462个蛋白中有276个蛋白被注释为分泌蛋白,192个蛋白被注释为细胞质膜蛋白(见图2c与图2d)。

**图2:**
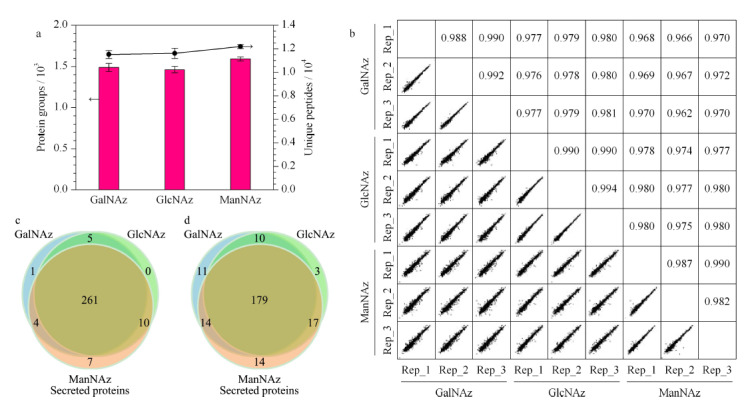
基于3种糖类似物的分泌蛋白质分析结果

我们也使用传统方法对无血清培养的HeLa细胞条件培养基进行了直接分析,3次重复实验中共鉴定到了1473个蛋白,其中249个为分泌蛋白。将传统方法与3种糖代谢标记方法鉴定到的分泌蛋白进行LFQ intensity的对比,可以看出,基于直接分析的传统方法,经过3次重复实验鉴定到的所有分泌蛋白的总LFQ强度(3次重复实验的平均值)显著低于代谢标记方法(见表1)。该结果表明,基于代谢标记的方式可以鉴定到更多的分泌蛋白。其中基于ManNAz的代谢标记在分泌蛋白质的富集中具有一定的优势,其富集到的分泌蛋白质数与细胞质膜蛋白质数多于其他两组,三者间的总体差异不大。共有的分泌蛋白质数为261个,在3组中的占比超过了90%;共有的细胞质膜蛋白数为179个,在3组中的占比超过了70%。

**表1:** 直接鉴定与基于代谢标记方式的富集结果

Enrichment method	Number of proteins	Number of secreted proteins	LFQ intensity
Direct enrichment	1473	249	4.38×10^10^
GalNAz	1487	271	1.04×10^11^
GlcNAz	1462	276	1.44×10^11^
ManNAz	1591	282	2.40×10^11^

LFQ: label-free quantification.

### 2.3 分泌蛋白的定量分析与糖基化位点鉴定分析

对3组糖类似物鉴定到的蛋白质进行进一步的无标定量分析。ManNAz组在分泌蛋白的定量富集中鉴定到的LFQ强度值相比于GalNAz组与GlcNAz组分别提高了130%与67.2%;在细胞质膜蛋白的定量富集中鉴定到的LFQ强度值相比于其他两组分别提高了273.3%与148.7%(见图3a)。三者在分泌蛋白和细胞质膜蛋白的定量富集中存在着显著的差异。分析其原因可能是因为分泌蛋白在进行糖基化修饰时通常更多地利用唾液酸进行修饰,而ManNAz在进入细胞后会转化为*N*-乙酰叠氮唾液酸,从而被有效地结合到分泌蛋白上。

**图3:**
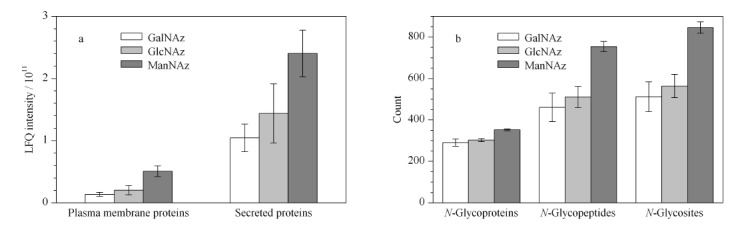
分泌蛋白的定量富集与糖基化鉴定结果(*n*=3)

图3b展示了3种糖类似物分别鉴定到的糖蛋白、糖肽以及糖基化位点的结果。ManNAz组鉴定到352个糖蛋白、754个糖肽以及846个糖基化位点;GalNAz组鉴定到290个糖蛋白、460个糖肽以及512个糖基化位点;GlcNAz组鉴定到302个糖蛋白、511个糖肽以及563个糖基化位点。ManNAz组的糖蛋白、糖肽与糖基化位点数目相比于GalNAz组分别提高了21.4%、63.9%以及65.2%;相比于GlcNAz组则分别提高了16.6%、47.6%以及50.3%。*N*-糖肽与*N*-糖基化位点的显著差异结果进一步表明ManNAz组定量富集分泌蛋白的优势可能来源于ManNAz对糖基化位点的高效标记。

### 2.4 分泌蛋白的差异化分析

对GalNAz、GlcNAz与ManNAz 3个实验组中鉴定到的分泌蛋白质进行差异化分析,其结果以火山图的形式表现。相比于GalNAz组,ManNAz组存在着1018个显著增加的蛋白与9个显著减少的蛋白,其中有141个被鉴定为显著增加的分泌蛋白(红点表示)(见图4a);相比于GlcNAz组,ManNAz组存在着121个显著增加的蛋白,其中有18个被鉴定为分泌蛋白(见图4b)。上述结果表明,ManNAz相比于GalNAz与GlcNAz具有更好的标记分泌蛋白的能力。图4c展示了GalNAz与GlcNAz之间的差异蛋白火山图,可以看出两种糖之间并无显著性差异的分泌蛋白,这可能是由于二者可以在细胞内相互转化,能够以相同的形式标记到蛋白质上,从而导致了它们的标记效率没有明显差异^[20,21]^。之后对图4中ManNAz组中显著增加的1018个蛋白做了GO分析。结果表明,这些差异化的蛋白主要分布在细胞外区域,与分泌蛋白的分布情况相符(见图4d)。上述结果表明ManNAz组相比于其他两组对分泌蛋白的定量富集具有高选择性。

**图4:**
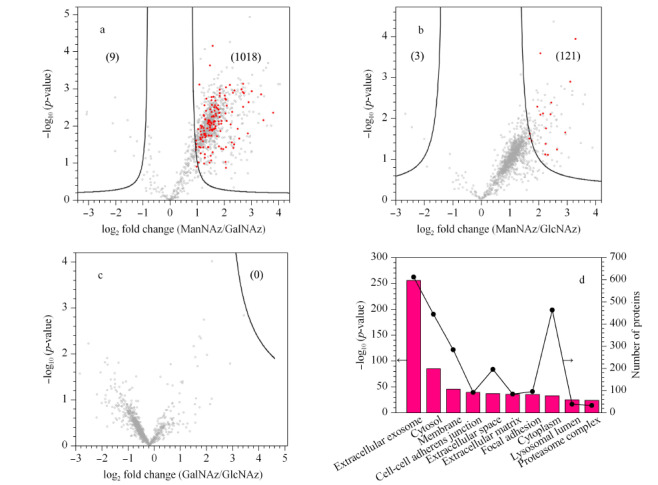
差异化蛋白分析

## 3 结论

分泌蛋白是许多疾病发生过程中的重要标志物,对于分泌蛋白的系统研究是目前的难点与热点。在这项工作中,我们将3种糖类似物(GalNAz、ManNAz和GlcNAz)进行了系统的比较,结合代谢标记、点击化学以及质谱检测对分泌蛋白进行了富集与分析。实验结果表明ManNAz对于分泌蛋白及相关蛋白的定量富集效果显著强于另外两种糖类似物。我们还发现基于ManNAz的代谢标记能鉴定到更多的糖基化位点,体现了ManNAz在分泌蛋白糖基化修饰过程中的巨大优势。本研究结果证明了ManNAz是研究基于糖代谢标记与点击化学相结合的分泌蛋白质组学的优秀工具,为分泌蛋白高选择性富集和系统分析提供了有益的对比分析和新技术策略。
